# P031: Room for improvement of clostridium difficile surveillance and reporting in denmark

**DOI:** 10.1186/2047-2994-2-S1-P31

**Published:** 2013-06-20

**Authors:** M Chaine, S Gubbels, E Tvenstrup Jensen, M Voldstedlund, K Mølbak, B Kristensen

**Affiliations:** 1 Microbiology and Infection Control, Copenhagen, Denmark; 2 Infectious Disease Epidemiology, Statens Serum Institut, Copenhagen, Denmark

## Introduction

National surveillance of Clostridium Difficile (CD) is currently performed in two ways in Denmark: by the Enteropathogen Registry (TBR) for all CD culture positive samples (reported from clinical microbiology laboratories), and since 2009 by a specific CD027 Registry with mandatory submission of CD strains for further characterization.

## Objectives

In this study the two systems are evaluated with a focus on completeness.

## Methods

From the TBR and the CD027 registry datasets were retrieved with data from January 1^st^ 2011 to December 31^st^ 2012 including information on date of sample and region of diagnostic microbiology department. For both, a patient was included only once during the observation period.

## Results

A total of 5342 patients were reported with CD in the TBR registry and 1971 patients in the CD027 registry. For the whole country the TBR showed a stable linear trend of percentages over the observation period with a coefficient of correlation (R^2^ ) of 0.0072, whereas the CD027 Registry showed a decreasing trend with R^2^= 0.52. Two regions accounted for a total of 1828 cases of CD027. Moreover, one of these regions recorded more cases in the CD027 registry than in the TBR in 19 of 24 months of the observation period.

## Conclusion

The decrease in number of cases in the CD027 registry may show a decrease in completeness as it coincided with known changes in laboratory practices. The observation that more cases from one of the regions were recorded in the CD027 Registry than in the TBR suggests an underreporting in the TBR register for this region. This shows there is a need for a surveillance system with higher completeness, which records ribotypes. The newly established Danish national microbiology database (MiBa), which includes all test results from all diagnostic microbiology departments, may provide the basis for such a system.

## Disclosure of interest

None declared

